# Correction to: Burden of Severe Illness Associated With Laboratory-Confirmed Influenza in Adults Aged 50–64 Years, 2010–2011 to 2016–2017

**DOI:** 10.1093/ofid/ofad045

**Published:** 2023-02-03

In the original publication of this article, [Kim P, Coleman B, C Kwong J et al. Burden of Severe Illness Associated With Laboratory-Confirmed Influenza in Adults Aged 50–64 Years, 2010–2011 to 2016–2017. Open Forum Infectious Diseases, Volume 10 Issue 1, January 2023. https://doi.org/10.1093/ofid/ofac664], IQVIA Solutions Canada Inc. was erroneously excluded from the Acknowledgments and Disclaimer sections and has been added to the version currently available online.

